# Factors influencing the match between patient needs and primary care services: Patient and healthcare provider perspectives from two Canadian provinces

**DOI:** 10.1177/13558196251405192

**Published:** 2025-12-01

**Authors:** Emilie Dufour, Maria Mathews, Emily Gard Marshall, Mylaine Breton, Jennifer E. Isenor, Dana Ryan

**Affiliations:** 1 Department of Family Medicine Primary Care Research Unit, 3688 Dalhousie University, Halifax, NS, Canada; 2 Department of Family Medicine, Schulich School of Medicine & Dentistry, 70384 Western University, London, ON, Canada; 3 Department of Community Health Science, Université de Sherbrooke, Longueuil, QC, Canada; 4 College of Pharmacy, 3688 Dalhousie University, Halifax, NS, Canada

**Keywords:** primary care, unattached patients, comprehensiveness

## Abstract

**Objectives:**

This study aims to combine the perspectives of patients and healthcare providers on the factors that influence the match between patients’ needs for and providers’ capacity to deliver services.

**Methods:**

A qualitative descriptive study design was used to examine perspectives and experiences of patients, family physicians and nurse practitioners in two jurisdictions in Canada: Nova Scotia and Ontario. Data from interviews were analyzed using the concept of comprehensiveness, the extent to which the approach to care and services delivered by providers meets patients’ needs, to examine how decisions were made in each province for patients to become members of a providers’ panel (the process known as ‘empanelment’).

**Results:**

Interviews with 33 providers and 25 patients were conducted in Nova Scotia and Ontario. Three areas of tension were identified in patients’ decision-making about choosing a regular primary care provider and providers’ decision-making about accepting regular patients. Patients and providers discussed the success of such a match as being influenced by personal characteristics, including attitudes, gender and cultural background, scope of practice and panel composition.

**Conclusions:**

Findings support the value of team-based care that considers the needs for empanelment of both patients and providers. This study provides healthcare providers with information to improve matching processes by incorporating these considerations (e.g., patient and provider sex, gender and chronic care needs) into decisions about empanelment. Mechanisms that reflect the characteristics and range of skills of the whole team rather than the family physician and nurse practitioner alone are an avenue for optimizing the success of empanelment.

## Introduction

In many countries, the patient experience with the healthcare system largely depends on their confirmed affiliation to a primary care provider, most often a family physician or, less frequently, a nurse practitioner.^1^ This formal attachment is known as empanelment in the Canadian healthcare system, defined as *‘*the deliberate process of assigning or linking a patient to a provider*’*, with the provider accepting responsibility for the patient.^1^ This is similar to the process of ‘registration’ with a general practice in the UK context, for example. Empanelment ensures a regular source of primary care, aims to secure a therapeutic relationship between the patient and a healthcare provider, and supports the management and planning of resources for the healthcare system.^2^ In Canada, family physicians and nurse practitioners typically have a panel (or list) of attached patients varying in size for which they have responsibility. The physicians and nurses may also provide access to an interdisciplinary team of healthcare professionals, depending on the practice model in which they operate.^3^ Family physicians may choose to have a focused practice, which involves delivering care to specific populations or in specific areas of clinical practice, such as palliative care and obstetrics.^4,5^

Formal attachment, or empanelment, can be achieved through various means. In Canada, attachment to nurse practitioners usually occurs through centralized waiting lists (CWLs). CWLs are used across all Canadian regions, although are applied in different ways. In Nova Scotia, for example, attachment to a primary care provider is on a first-come, first-served basis, while some other provinces, including Quebec and Ontario, apply a system whereby patients are prioritized according to the assessment of their complexity or vulnerability.^3^ Given current physician shortages and long waiting times for attachment, patients may feel that they have limited ability to choose their regular primary care provider.

The means by which providers attach patients have been studied mostly with physicians. Their participation in CWLs is voluntary. Physicians can attach family members of existing patients, or by other means, including by enrolling new patients of their own choosing. In some cases, family physicians use interviews, also known as “meet and greets”, for which Smith and Marshall^6^ suggested four typologies, including one with a rationale of assessing the alignment between patient needs and provider scope. Family physicians can refuse patients, for instance if they consider that the patient’s needs are not compatible with their scope of practice.^7^

Attachment processes are an important determinant of comprehensiveness, but are rarely considered from this perspective. Comprehensiveness refers to the extent to which primary care services meet most of a patient’s healthcare needs.^8^ The concept includes: (1) the approach to care, focusing on the recognition of a wide range of needs, including biomedical, psychological and sociocultural, and (2) the provision of a broad range of services including promotion, prevention, treatment and rehabilitation to manage a wide spectrum of health conditions.^9^

Previous studies have underlined variations in comprehensiveness based on differences in the range of services delivered by family physicians and their scope of practice.^4,10^ Other studies have focused on the expectations of patients towards their regular primary care provider.^10–17^ This paper aims to combine the perspectives of patients and providers on the factors that influence the match between patients’ needs and the providers’ capacity to deliver services that meet those needs. The objective of this study is to answer the following question: “What factors promote and hinder a successful match between patients’ needs and primary care providers’ practice?”

## Materials and method

The method is reported according to the criteria for reporting qualitative research.^18^ We used a qualitative descriptive study design^19^ to examine the perspectives of patients and providers on the factors that influence the match between the patients’ needs and the providers’ capacity to deliver needed services in two jurisdictions in Canada, i.e., Nova Scotia and Ontario. In both regions, patients can be formally attached to a family physician or a nurse practitioner as their regular provider. Primary care is delivered through different practice models in both regions, including team-based and solo practices.^3^ This study is part of a larger mixed-methods study that aimed to examine the effectiveness of centralized waiting lists across different Canadian regions, as well as to describe the experiences and perspectives of providers and patients related to patient attachment, including access to providers, and the means and criteria used for empanelment.^20^

We used purposive and snowball sampling methods to recruit family physician and nurse practitioner participants. Purposive sampling ensured that diverse participant characteristics such as profession (i.e., family physician or nurse practitioner), gender and region (e.g., urban/rural, health zone), were represented. Potential participants were identified by researchers, the research team’s network of primary care stakeholders, and other participants (i.e., snowball sampling). We relied upon partner organizations (physician and nursing professional associations, relevant university departments, primary care clinics) and social media (Facebook and Twitter) to distribute invitations to potential participants. Eligible provider participants had to be in active primary care practice in either Ontariso or Nova Scotia at the time of the interview.

A variety of methods were used to recruit patients: invitations were posted on social media platforms (Facebook pages dedicated to finding doctors and new residents and Twitter), with community associations, and through snowball sampling. We included patients 18 and older who were unattached or had recently found a regular primary care provider at the time of the interview. Patients had to be residents of the province in which they were interviewed. We excluded students (many of whom retain residency and health insurance coverage from their home province), temporary foreign workers, and tourists. Interested participants were asked to contact the research coordinator in their respective province. We informed the participants about the objectives of the mixed-method study.

A multidisciplinary team developed the interview guides using findings from a policy scan and analysis and input from members of the study team, which included representatives from various interest-holder groups, including clinicians and policymakers. The interviews were conducted by trained research assistants with extensive experience in conducting qualitative interviews. The interviews were conducted in English via Zoom (Zoom Video Communications Inc.) or telephone, depending on participant preference. Provider interviews took place between October 2020 and July 2021, and patient interviews between May 2021 and May 2022. We audio-recorded interviews and transcribed them verbatim.

The research team determined that thematic saturation had been reached when no new themes were identified.^21^ We managed data using NVivo software (QSR International Pty Ltd, 2018). We analyzed transcripts and field notes taken by the interviewer that documented observations and identified themes using thematic analysis.^22^ In each province, we developed a coding template by having two members of the research team independently read initial transcripts to identify recurring ideas and themes. Researchers from both provinces met to compare themes and ensure that there was a consistent understanding. This process was overseen by the principal investigators. Any disagreements were resolved through discussion and codes were adjusted to increase clarity until a harmonized coding template was created across both provinces. This coding template was then used to code all the transcripts. In this paper, we used the concept of comprehensiveness to examine the data from each province relating to how decisions were made to accept the attachment or patient into practice.

## Results

A total of 58 interviews were conducted in Nova Scotia (*n* = 42) and Ontario (*n* = 16), including 33 with providers and 25 with patients (Table 1). Participants are anonymised and allocated a reference number in the extracts from interviews quoted below, with patients referred to as PT1-25, Family Physicians (FP) and Nurse Practitioners (NP). Locations are abbreviated to ON (Ontario) and NS (Nova Scotia). We identified three areas of tension in patients’ decision-making about choosing a regular primary care provider and providers’ decision-making about accepting regular patients: (1) personal characteristics, (2) provider scope of practice, and (3) panel composition (Table 2).

**Table 1.** Characteristics of participants.

	Nova Scotia patients (*n* = 15)	Nova Scotia providers (*n* = 27)	Ontario patients (*n* = 10)	Ontario providers (*n* = 6)	Total (*n* = 58)
Type of provider					
FP	N/A	20 (74)	N/A	4 (66.6)	24 (72.7)^a^
NP	N/A	7 (26)	N/A	2 (33.3)	9 (27.3)^a^
Gender^b^					
Men	2 (13.3)	9 (33.3)	3 (30)	2 (33.3)	16 (27.6)
Women	12 (80)	18 (66.6)	7 (70)	4 (66.6)	41 (70.7)
Gender fluid	1 (6.7)	0 (0)	0 (0)	0 (0)	1 (1.7)
Age					
25–49	9 (60)	N/A	6 (60)	N/A	15 (60)^c^
50–64	4 (26.7)	N/A	4 (40)	N/A	8 (32)^c^
65+	2 (13.3)	N/A	0 (0)	N/A	2 (8)^c^
Years in practice	N/A	13.4 (±8.7)	N/A	9.6 (±13.3)	9.5 (±12.7)^a^
Current community					
Rural	5 (33.3)	13 (48.1)	0 (0)	0 (0)	18 (31)
Urban	10 (66.7)	14 (51.9)	10 (100)	6 (100)	40 (69)

^a^Based on total number of providers (*n* = 33).

^b^Gender was asked as an open-ended question.

^c^Based on total number of patients (*n* = 25).

**Table 2.** Overview of the main areas of tension in decision-making for empanelment.

Research findings	Areas of tension		
Theme	**Personal characteristics**	**Provider type and scope of practice**	**Panel composition**
Key concepts	Cultural competency	Benefits of having specific expertise	Seeking diversity in practice
	Gender sensitivity	Need for access to a wide range of services	Balancing the complexity of care
	Match in personality	Differences in perception of scope of practice between FPs and NPs	Fairness to other providers

## Personal characteristics

Participants discussed personal characteristics that influenced their relationship with the primary care provider and that could have an impact on the success of the match with the provider. For example, patients in this study discussed the benefits of having a regular provider who has an understanding of their cultural background and gender:

“Ideally cultural competency. [… ] I know there are also people that would love to see someone that spoke their language and not have to worry about experiencing racism from their doctor.” [PT8, NS].

Some participants felt that the fit between the provider’s approach and individual patient characteristics was even more important to consider for specific populations:

… I think especially if you’re thinking about marginalized patients, they should have so much control over who their provider is. I think, just like trans folks and people of color, that’s really important. [… ] people who’ve experienced trauma, I just want them to have total control. And I think that’s where it’s hard in places where it’s just whoever it is, there’s nothing you can really do about it. [PT7, ON]

Patients reported that gender was not a factor for a successful match with their regular provider, stating

“Honestly, man or woman doctor, or whoever, I don’t care.” [PT1, NS].

Some providers, on the other hand, felt that their gender as such was an issue for some patients and that it was a source of mismatch, noting that patients may say

“No, I don’t want a male doctor”. [FP3, NS].

However, patients did expect their regular provider to be able to provide gender-specific care to some extent, including the recognition of gender-related needs:

Definitely being young and definitely being female presenting. It’s well documented that my pain is not considered as serious based on my own self-report. […] You see it in the length it takes to diagnose things like endometriosis, and how that’s treated. And you see it in how people are treated when they go to the hospital. And it’s resulted in death. [PT7, NS]

Some patients specified that they did not expect a single provider to match all their preferences, as long as there was availability and variety among the team:

Any practice that I’ve been a part of you do have that choice [of provider]. If there isn’t a female physician in the practice, then you can have a nurse come in or a staff member of your choosing. Anyone that makes you feel comfortable. […] They do try to protect both parties upon your level of comfort. I haven’t found that difficult to request or to navigate in any physician’s office in Nova Scotia or in Ontario. [PT2, NS]

Having a provider who takes the time to explore different avenues for treatments and is open minded when discussing health conditions was a characteristic that was highly valued by patients in this study:

“[My regular provider] is really open to trying things that are not medicine right away. Which I love. He’s really open about everything.” [PT2, NS].

Patients also discussed how the personality fit was an important factor in the matching success, as it impacted how comfortable they were with them in discussing their health:

In terms of building that respect and the trust that you need between each other, it has to be a good match. […] If you get a physician that isn’t connecting with you in the way that you need a physician to, you’re probably not going to be as open about some of the challenges that you’re having or even seek care because you’re not going to feel confident in the relationship that you have with that person. So that’s problematic as well. […] We [sometimes hold] physicians to different standards, and we forget that they are people, too. [PT2, NS]

Matching in terms of personality was also mentioned by some providers as a factor likely to influence their willingness to take on patients. However, this criterion was applied mainly in specific situations, for example, where they felt they had achieved a full workload:

Most of the patients that I take on, it’s because they’re a family member and I think that they’re reasonable people and they’re nice people. And I don’t mind saying that because, I feel like I’m at my capacity, so I feel like if I am taking anyone else, I would like it to be someone that’s nice and not going to cause trouble. Not cause trouble but cause more stress than the average person. I don’t care about age, I’m not a like, ‘only seeing kids and babies person’, I'm not all geriatrics, it doesn’t matter to me, to be honest. It’s just more of knowing the immediate family. [FP4, NS]

## Provider type and scope of practice

Patients and providers agreed that the providers’ range of skills significantly impacted the match between the needs and care provided. Some patients did not want a nurse practitioner as a primary care provider, believing that they had a limited scope of practice:

“Because a lot of people will get a call and say you’ve been matched with a nurse practitioner, and they say, “No, I don’t want a nurse practitioner. I want a doctor.” [FP3, NS].

For patients, the preference for a family physician as their usual provider was driven by a concern to ensure access to a wide range of services, as they believed that, in some cases, the scope of services provided by nurse practitioners was limited:

I was under the impression that they could prescribe certain medications. I don’t feel like it's a function of nurse practitioners not being competent so much as it’s a licensing issue of what they’re allowed to do. […] I feel like there’s a lot of underutilized medical experience that because you're not [a physician], you’re not allowed to do certain things. [PT7, NS]

Patients discussed a range of needs they expected their provider to be able to address, including the provision of psychological and biomedical care. Mental health and women’s health were often mentioned by patients as domains in which there were variations in the level of involvement and depth that primary care providers were able to manage. Some patients felt that these domains required advanced skills:

“There’s a lot to be learned about [women’s health] … And especially in family practice where it’s so broad. […] Just to focus on these women’s issues that are so important.” [PT1, NS].

Others expressed disappointment that their regular provider was unable to offer services that they felt should be delivered in primary care and should not require specialist referral:

I would have loved to be able to say I would like a doctor that is more skilled or comfortable with mental health than not. Or that can do a pap or can insert [intrauterine devices]. Because not everyone will or has that expertise. So, that would save me having to get referred out for a bunch of things. Which seems like it would be more efficient. [PT8, NS]

On the other hand, some patients felt that expecting their regular provider to be able to meet all their healthcare needs sometimes led to them managing care that patients considered to be outside their area of expertise:

I think that we ask a lot of our [family physicians] and some of them have a sense that they can resolve any health issue you have and they’re reluctant to send you to a specialist. So, then you get really poor care. [PT1, ON]

Providers had mixed feelings about the range of services they should provide. Both family physicians and nurse practitioners valued being comfortable with services they provide, while recognizing that this could create a tension with the needs of the broad range of patients, they should be able to respond to:

I think you need to have a comfort level with what you’re doing. For a provider to take on a whole bunch of female patients of childbearing age, if they’re really not comfortable with providing prenatal care, that’s not a great thing for them to do. I don't think that they should be able to exclude them entirely, but maybe not have that as a focus, whereas somebody else who maybe, as part of their practice, provides obstetrical care, that’s a really good fit for both that patient and the provider. So, I do get a little bit of wanting to have a focus to your practice, but not at the detriment of other patients. [NP2, ON]

Establishing a focus or specialization in their practice was inevitable for some providers, but ways in which these areas of expertise were developed varied among them. Family physicians considered the wide range of services to be covered by a provider could sometimes be incompatible with the specific degree of involvement or speciality required in certain domains of health care. Some providers focused their practice on their specific interests, while for others, their areas of expertise had developed in response to their patients’ needs:

… no matter how much you try to remain a generalist, you develop a niche just by following the needs of your patients. I’m much more likely to attend Continuing Medical Education that I think are going to benefit more of my patients as opposed to things that might only come up once every couple of years. [FP7, NS]

Defining a focus in practice was often viewed as essential to the delivery of high-value healthcare given the growing range of treatment options and patient complexity. Some providers felt that it was unrealistic to expect physicians to be skilled in the overall range of primary care services required to address patients’ needs. A physician from Ontario described how focused knowledge and practice will be integral to the future of primary care:

I think that’s the way family medicine is going to evolve [on focused knowledge and practice] whether you like it or not, whether you think we should be training holistic family doctors to give them a tiny bit of knowledge about a lot, a lot of knowledge about nothing, and I think they’re totally intimidated; they’re scared to practice because they don't have enough knowledge and experience. So, creating opportunities like these focused practice, is I think, a great idea. [FP1, ON]

From providers’ perspectives, working in collaborative practices was a way to better address patients’ health care needs, and to have more confidence in their capacity to care for patients with needs they felt less comfortable responding to:

Family physicians [might be] overwhelmed with treating patients from a knowledge perspective on multimorbidity. […] The solo family physician knows their boundaries of being able to provide care and a very complex patient might need a team, and they might not have that team and so, feel worried [if can they best] serve that patient. [FP5, ON]

## Panel composition

Providers’ caseload composition, i.e., patients formally attached to them, and the criteria according to which they take on patients into their practice, have an important influence on the matching process from a population perspective. Providers emphasized that diversity is often one of the reasons they chose to work in primary care. Therefore, they often valued a diverse caseload, both in terms of demographics and range of conditions they care for:

… for some practitioners having a diversity in their practice is important. That be it, based on ethnicity, based on age, based on you know, socioeconomic status. […] Because potentially these are the people you’ll be working with for the rest of your life. So, I think there is a value to being able to, with the intention of diversity to be able to select characteristics. [FP19, NS]

Patients were aware that their primary care provider usually sought balance in the characteristics of patients they cared for, and that this could have an impact on their choice to manage certain populations:

The other thing to consider is, they probably want to balance their practice with some people who, their needs are really complicated and some people where their needs are not very complicated. And I think part of the joy of family medicine is that you get a lot of variety, so I imagine that is important to them. [PT7, ON]

From providers’ perspective, a balance in their caseload was important to prevent exhaustion and burnout. This was a factor they considered when accepting patients in their practice. However, providers found the system unlikely to prevent providers from cherry-picking, and that selecting patients based on certain criteria could be an interesting way of avoiding heavy workloads:

Human nature being what it is, to make the same amount of money for less work is an obvious draw for some people. But I think that fails to take into account why most of us went into medicine in the first place, which is to help those in need regardless of the level of care need. But if you had an entire roster full of medically complex patients that took a lot of time, took a lot of energy, emotional or otherwise, that’s also not good for someone long term. So I think you should be able to have a mix of both. [FP20, NS]

Some participants suggested that providers might be reluctant to having a focused practice on specific populations, worrying that it might create a work overload:

…I think one of the barriers that patients who are immigrants perceive is that it’s sometimes difficult to see a provider who’s maybe not as comfortable providing care to immigrants. It may improve access if physicians who have an expertise or comfort level with a certain group of patients, or a certain population, are getting more of those patients. But then, I think again, the trade-off is that that may end up with an unfair distribution of patients to providers. [FP6, ON]

Providers also emphasized the benefits of a focused practice for addressing comprehensive healthcare needs, emphasizing that a focused practice should be directed to populations with more specific or complex needs:

The double-edged side of [following specific populations] is, should a physician say, “I only want a 20- to 30-year-old with no comorbidities that are healthy,” no, I don’t think that’s fair. I think if used with appropriate checks and balances, it may not be unreasonable to optimize physicians to have a specialized expertise for a focused population that would require your care. [FP10, NS]

One nurse practitioner in Nova Scotia also noted the need to balance the workload among providers in a spirit of fairness:

I think it’s nice in practice to have a good mixture of healthy adults, young babies, elderly. But I wouldn’t advocate for preferentially doing that because say I decided I wanted all babies. That means somebody is going to get all really elderly. We’re all being paid the same. [NP21, NS]

The lack of mechanisms to ensure proper guidance in balancing the caseload of providers were raised by several participants, which increased the risk of unintended consequences:

For someone to say, “Oh, I have expertise in diabetes; I want to roster only diabetic patients,” in an otherwise, normal capitation model practice, that seems unfair. It’s providing benefit to yourself in ways that don’t necessarily provide a benefit for patients, and I would be a bit uncomfortable with that. It’s a tough line because there’s room for interpretation at each one of those processes, and I don’t trust doctors to be able to make that decision for themselves in a way that won’t advantage themselves. [FP4, ON]

Patients discussed how a focused practice could support better access to care for specific populations:

If a family doctor has a passion about a certain area of care and they would like to see all patients with those types of conditions and get really good at managing them, I feel like that could be good for those patients. [PT5, ON]

## Discussion

We aimed to explore factors influencing the match between patients’ needs and the care provided by family physicians and nurse practitioners. Patients and providers discussed the success of such a match as being influenced by personal characteristics, including attitudes, gender and cultural background, scope of practice and panel composition.

Patients in this study valued comprehensiveness both in terms of the range of conditions their regular provider was able to address, and in terms of the depth of expertise to manage conditions. Findings from this study show that patients have a variety of needs, and that the qualities they value most in their provider therefore differ according to their individual needs. A systematic review by Pratiwi et al.^10^ on patients’ values regarding primary care, emphasized the importance for patients to have a provider that is inclusive in their approach, notably in terms of gender, ethnicity, socioeconomic status and mental health. Those findings echo the characteristics and domains of care addressed by patients in our study. The study by Leach et al.^16^ reported that patients overall had a preference for physicians in terms of qualifications and for nurse practitioners in terms of interpersonal skills as their primary provider. Other studies have reported that patients first prioritized quality of care, followed by communication skills.^13,15^

Patients in this study generally did not expect their regular individual provider to be able to meet all their needs but generally expected their clinic to be able to deliver comprehensive care, namely care consistent with their personal characteristics and preferences, and services covering their overall healthcare needs. These findings partly mirror those of previous studies, which report patients’ expectations as relying almost exclusively on their family physician.^10,13,16^ Physician-only dependence is not sustainable for comprehensiveness in primary care.^23^ Some physicians in our study noted that patients were sometimes reluctant to be attached to a nurse practitioner. The attitude of physicians is critical to the capacity of primary care to move towards an integrated team-based approach, both in their openness to redistributing work and in promoting with patients their confidence in the capacity of other clinicians to meet healthcare needs.^23,24^ Based on these findings, we recommend that more evidence be generated on the effectiveness of nurse practitioners as primary care providers, and that this workforce be further promoted to the wider patient population to improve understanding and comfort. Building patient confidence in nurse practitioners requires effective public communication and improved patient education on the management of healthcare needs by non-medical primary care providers, as well as implementation of policies such as systems to support practice-based triage.^25^

From a provider’s perspective, results in our study highlight the challenges of having expertise in the in-depth management of all patient needs, including biopsychosocial ones. These findings are consistent with previous studies that reported the challenges for physicians to have a comprehensive practice.^4,26^ Factors such as changes in complexity of care and aging populations, administrative burden and other duties in hospitals, long-term care facilities and emergency departments, further increase pressure on primary care physicians.^4^ Patients and providers in our study noted benefits of a focused practice similar to those emphasized in previous studies, while raising important challenges in meeting patient needs from a population-based perspective and fairness among primary care providers.^5^ Some participants also emphasized the importance of managing patients with diverse needs and characteristics as a core component of family medicine, which has also been reported by previous studies.^26^

Findings from our study suggest that clinicians who share the same profession may have very different perceptions of their capacity and comfort level in terms of the range of services they can provide, and on the benefits of having a focused practice. These findings point to the need to consider more closely the individual needs and capacity of family physicians and nurse practitioners in the organization of primary care services. Team-based primary care is the preferred practice model for ensuring a high level of comprehensive care.^24^ This model is most often addressed in terms of interprofessional collaboration and considers to a much lesser extent the complementarity between providers who share the same profession within the same setting.^27,28^ Considering the differences in the scope of practice, personal characteristics that influence the relationship with patients, and the level of comfort required to perform certain services, we recommend that greater emphasis be placed on staff composition both from an intra- and inter-professional perspective when addressing skill mix. Skill mix and staff patterns in primary care could aim to ensure complementary expertise in focused practice in service areas where there are gaps, in addition to their general practice including general services, including routine follow-ups and management of chronic conditions.

In terms of empanelment, providers in this study often considered the attachment of new patients to their practice based on the impact on their current caseload. This factor can be detrimental to populations with complex needs.^29^ These findings have implications for the means through which patients are empaneled to primary care practices. In this study, the range of services available to patients largely depended on the provider they were attached to, which reflects the primary care model for empanelment in Canada. Even when patients’ regular provider was within a team-based practice, the matching process focuses on a single provider, either the family physician, or, to a lesser extent, the nurse practitioner. While the benefits of empanelment to a single provider process have been highlighted, this process can create challenges from a comprehensiveness perspective, as it is based on the capacity of a single provider, rather than that on the whole team. Empanelment to a single provider is likely to reinforce the assumption of sole responsibility of the regular provider, as there is no formalization of shared responsibility. Exploring ways to empanel patients in a way that formalize the team’s shared responsibility, contributions and skill mix could be an avenue to provide more capacity from a comprehensive perspective. The role of panel manager^23,24,30^ in a team-based rather than single-provider empanelment could be further explored with regard to ensuring continuity and coordination, without placing responsibility for the patient solely on the healthcare provider.

### Limitations

The findings in this study may not capture the experiences and perspectives of patients and providers on the factors influencing the matching process in other regions of Canada. The number of nurse practitioners (*n* = 9) included in this study is lower than that of family physicians (*n* = 24), which is consistent with the higher number of family physicians in Canada, but does not mirror the proportion between Ontario and Nova Scotia. The small number of nurse practitioners limited opportunities to contrast perspectives by type of provider. The methods used for sampling may have introduced selection bias, as participants who are part of professional and social networks may be more engaged or connected, and recruitment via social media excludes people who are not digitally connected. These factors may affect diversity and generalisibility of the findings. In addition, our results are limited by the non-inclusion of administrative staff in the sample, who may play an important role in translating patients’ needs and in their knowledge of the system and communities.^31^

## Conclusion

Through qualitative interviews with both patients and primary care providers, we identified three areas of tension that patients considered in choosing a regular care provider and providers considered in taking on a regular patient: (1) personal characteristics, (2) provider scope of practice, and (3) panel composition. These areas of tension influence providers’ ability to manage certain patients, and the extent to which patients feel their needs are being adequately met. Patients did not expect their regular provider to be able to meet all of their needs but generally expected their clinic to be able to deliver comprehensive care, namely care that is consistent with their personal characteristics and preferences, and services covering their overall healthcare needs. Providers have very different perceptions of their capacity and comfort level in terms of the range of services they can provide patients and the need to balance expertise in a specific area with the ability to maintain competence in a broad range of skills. Our findings support the need for team-based care, that addresses the paneling decisions needs of both patients and providers. The findings also provide healthcare providers, including centralized waitlist managers, with information to improve matching process by incorporating these considerations (e.g., patient and provider sex and gender and chronic care needs) into the intake forms. Mechanisms that reflect the characteristics, range of skills and panel composition of the whole team rather than family physicians and nurse practitioners could be a further avenue for optimizing the success of attachment.

## Data Availability

The datasets generated during and/or analyzed during the current study are available from the corresponding author on reasonable request.*

## References

[1] Aggarwal M Glazier RH . Toward a universal definition of provider-patient attachment in primary care. Can Fam Physician 2024; 70: 634–641. 39406419 10.46747/cfp.7010634 PMC11477241

[2] Breton M Smithman MA Kreindler SA , et al. Designing centralized waiting lists for attachment to a primary care provider: considerations from a logic analysis. Eval Progr Plann 2021; 89: 101962. 10.1016/j.evalprogplan.2021.101962 34127272

[3] Breton M Wong ST Smithman MA , et al. Centralized waiting lists for unattached patients in primary care: learning from an intervention implemented in seven Canadian provinces. Healthc Policy 2018; 13: 65–82. 30052190 10.12927/hcpol.2018.25493 PMC6044259

[4] Lavergne MR Rudoler D Peterson S , et al. Changes in comprehensiveness of services delivered by Canadian family physicians: analysis of population-based linked data in 4 provinces. Can Fam Physician 2023; 69: 550–556. 37582603 10.46747/cfp.6908550 PMC10426375

[5] Kabir M Randall E Mitra G , et al. Resident and early-career family physicians’ focused practice choices in Canada: a qualitative study. Br J Gen Pract 2022; 72: e334–e341. 35023851 10.3399/BJGP.2021.0512 PMC8936180

[6] Smith V Marshall EG . “Meet and greets” in family practice. Can Fam Physician 2021; 67: e227–e234. 34385217 10.46747/cfp.6708e227 PMC9683423

[7] Smithman MA Haggerty J Gaboury I , et al. Improved access to and continuity of primary care after attachment to a family physician: longitudinal cohort study on centralized waiting lists for unattached patients in Quebec, Canada. BMC Prim Care 2022; 23: 238. 36114464 10.1186/s12875-022-01850-4 PMC9482231

[8] Henry TL Rich EC Bazemore A . Comprehensiveness—The need to resurrect a sagging pillar of primary care. J Gen Intern Med 2022; 37: 229–231. 34346009 10.1007/s11606-021-07071-2 PMC8330818

[9] Grudniewicz A Randall E Jones L , et al. Comprehensiveness in primary care: a scoping review. Milbank Q 2025; 103: 153–204. 39671532 10.1111/1468-0009.12723 PMC11923724

[10] Pratiwi AB Padmawati RS Mulyanto J , et al. Patients values regarding primary health care: a systematic review of qualitative and quantitative evidence. BMC Health Serv Res 2023; 23: 400. 37098522 10.1186/s12913-023-09394-8 PMC10131468

[11] Lim AH Ng SW Teh XR , et al. Conjoint analyses of patients’ preferences for primary care: a systematic review. BMC Prim Care 2022; 23: 234. 36085032 10.1186/s12875-022-01822-8 PMC9463739

[12] Wun YT Lam TP Lam KF , et al. How do patients choose their doctors for primary care in a free market? J Eval Clin Pract 2010; 16: 1215–1220. 20695952 10.1111/j.1365-2753.2009.01297.x

[13] Mercado F Mercado M Myers N , et al. Patient preferences in choosing a primary care physician. J Prim Care Community Health 2012; 3: 125–131. 23803456 10.1177/2150131911421802

[14] Khatami F Shariati M Khedmat L , et al. Patients’ preferences in selecting family physician in primary health centers: a qualitative-quantitative approach. BMC Fam Pract 2020; 21: 107. 32527224 10.1186/s12875-020-01181-2 PMC7291526

[15] Dahlgren C Dackehag M Wändell P , et al. Simply the best? The impact of quality on choice of primary healthcare provider in Sweden. Health Policy 2021; 125: 1448–1454. 34645569 10.1016/j.healthpol.2021.09.009

[16] Leach B Gradison M Morgan P , et al. Patient preference in primary care provider type. Healthc 2018; 6: 13–16. 10.1016/j.hjdsi.2017.01.001 28602803

[17] Bornstein BH Marcus D Cassidy W . Choosing a doctor: an exploratory study of factors influencing patients’ choice of a primary care doctor. J Eval Clin Pract 2000; 6: 255–262. 11083036 10.1046/j.1365-2753.2000.00256.x

[18] O’Brien BC Harris IB Beckman TJ , et al. Standards for reporting qualitative research: a synthesis of recommendations. Acad Med 2014; 89: 1245–1251. 24979285 10.1097/ACM.0000000000000388

[19] Doyle L McCabe C Keogh B , et al. An overview of the qualitative descriptive design within nursing research. J Res Nurs 2019; 25: 443–455. 34394658 10.1177/1744987119880234 PMC7932381

[20] Marshall EG Breton M Green M , et al. CUP study: protocol for a comparative analysis of centralised waitlist effectiveness, policies and innovations for connecting unattached patients to primary care providers. BMJ Open 2022; 12: e049686. 10.1136/bmjopen-2021-049686 PMC8905966 35256440

[21] Guest G Namey E Chen M . A simple method to assess and report thematic saturation in qualitative research. PLoS One 2020; 15: e0232076. 32369511 10.1371/journal.pone.0232076 PMC7200005

[22] Braun V Clarke V . Using thematic analysis in psychology. Qual Res Psychol 2006; 3: 77–101.

[23] Bodenheimer TS Smith MD . Primary care: proposed solutions to the physician shortage without training more physicians. Health Aff 2013; 32: 1881–1886. 10.1377/hlthaff.2013.0234 24191075

[24] Bodenheimer T Ghorob A Willard-Grace R , et al. The 10 building blocks of high-performing primary care. Ann Fam Med 2014; 12: 166–171. 24615313 10.1370/afm.1616 PMC3948764

[25] Ranerup A Norén L Sparud-Lundin C . Decision support systems for choosing a primary health care provider in Sweden. Patient Educ Counsel 2012; 86: 342–347. 10.1016/j.pec.2011.06.013 21778027

[26] Grudniewicz A Randall E Lavergne MR , et al. Factors influencing practice choices of early-career family physicians in Canada: a qualitative interview study. Hum Resour Health 2023; 21: 84. 37884968 10.1186/s12960-023-00867-9 PMC10605974

[27] Wagner EH Flinter M Hsu C , et al. Effective team-based primary care: observations from innovative practices. BMC Fam Pract 2017; 18: 13. 28148227 10.1186/s12875-017-0590-8 PMC5289007

[28] Swankoski KE Peikes DN Palakal M , et al. Primary care practice transformation introduces different staff roles. Ann Fam Med 2020; 18: 227–234. 32393558 10.1370/afm.2515 PMC7213997

[29] Lin J Bates S Allen LN , et al. Uptake of patient enrolment in primary care and associated factors: a systematic review and meta-analysis. BMC Prim Care 2025; 26: 76. 40119278 10.1186/s12875-025-02779-0 PMC11927268

[30] Altschuler J Margolius D Bodenheimer T , et al. Estimating a reasonable patient panel size for primary care physicians with team-based task delegation. Ann Fam Med 2012; 10: 396–400. 22966102 10.1370/afm.1400 PMC3438206

[31] Dakin F Rai T Paparini S , et al. Supporting your support staff during crises: recommendations for practice leaders to develop a relational workplace. BMJ Lead 2023; 7: 1.1–7.

